# Wnt signalling induces accumulation of phosphorylated β-catenin in two distinct cytosolic complexes

**DOI:** 10.1098/rsob.140120

**Published:** 2014-11-12

**Authors:** Jan P. Gerlach, Benjamin L. Emmink, Hisashi Nojima, Onno Kranenburg, Madelon M. Maurice

**Affiliations:** 1 Department of Cell Biology, Center for Molecular Medicine, Heidelberglaan 100, Utrecht 3584CX, The Netherlands; 2 Department of Surgery, University Medical Center Utrecht, Heidelberglaan 100, Utrecht 3584CX, The Netherlands; 3 MRC National Institute for Medical Research, The Ridgeway, Mill Hill, London NW7 1AA, UK

**Keywords:** Wnt signalling, β-catenin, colon cancer, protein complexes, Axin1, blue native/SDS-PAGE

## Abstract

Wnt/β-catenin signalling controls development and adult tissue homeostasis and causes cancer when inappropriately activated. In unstimulated cells, an Axin1-centred multi-protein complex phosphorylates the transcriptional co-activator β-catenin, marking it for degradation. Wnt signalling antagonizes β-catenin proteolysis, leading to its accumulation and target gene expression. How Wnt stimulation alters the size distribution, composition and activity of endogenous Axin1 complexes remains poorly understood. Here, we employed two-dimensional blue native/SDS-PAGE to analyse endogenous Axin1 and β-catenin complexes during Wnt signalling. We show that the size range of Axin1 complexes is conserved between species and remains largely unaffected by Wnt stimulation. We detect a striking Wnt-dependent, cytosolic accumulation of both non-phosphorylated and phosphorylated β-catenin within a 450 kDa Axin1-based complex and in a distinct, Axin1-free complex of 200 kDa. These results argue that during Wnt stimulation, phosphorylated β-catenin is released from the Axin1 complex but fails to undergo immediate degradation. Importantly, in APC-mutant cancer cells, the distribution of Axin1 and β-catenin complexes strongly resembles that of Wnt-stimulated cells. Our findings argue that Wnt signals and APC mutations interfere with the turnover of phosphorylated β-catenin. Furthermore, our results suggest that the accumulation of small-sized β-catenin complexes may serve as an indicator of Wnt pathway activity in primary cancer cells.

## 2. Introduction

Wnt/β-catenin signalling orchestrates stem cell maintenance, proliferation and cell-fate decisions during embryonic development and in adult tissue homeostasis. Central to the Wnt/β-catenin signalling cascade is the proteolytic regulation of β-catenin, which performs a dual role in cell adhesion and transcriptional activation. In epithelial cells, β-catenin is constitutively synthesized to allow its stable integration in adherens junctions at the plasma membrane [1]. In the absence of Wnt ligands, the level of the unbound, cytosolic pool of β-catenin is kept very low through the activity of a multi-protein complex dedicated to degrade β-catenin [2,3]. Formation of this β-catenin destruction complex is coordinated by the scaffold protein Axin1, which recruits the kinases CK1 and GSK3β and the tumour-suppressor protein APC [3–8]. The complex binds and phosphorylates cytosolic β-catenin at its flexible N-terminus, after which the protein is degraded by the ubiquitin–proteasome system [2,3,7].

Wnt binding to its receptors Frizzled and LRP5/6 at the plasma membrane inhibits the degradation of β-catenin. As a consequence, β-catenin accumulates and migrates to the nucleus to bind its partner TCF/LEF and drive expression of Wnt target genes [9,10]. The mechanism by which the activated Wnt receptor complex inhibits β-catenin degradation is a subject of intense debate. A key signalling step is the assembly of a multimeric signalosome, comprising Frizzled/LRP6, the effector protein Dishevelled and the Axin1-based destruction complex [11–19]. The molecular steps by which Wnt-activated receptors control β-catenin stabilization, however, remain enigmatic. A shared view in current models is that β-catenin degradation is inhibited at the level of the destruction complex, either through inhibition of GSK3β [20–22], sequestering of GSK3β by multi-vesicular bodies [23], disassembly of the Axin1 complex [24–26], impaired β-catenin binding [14,27], inhibition of β-catenin ubiquitination [28], or a combination of these events.

The scaffold protein Axin1 is essential in the assembly of both the destruction complex and the Wnt-induced signalosome, and thus serves a key role in the regulation of the ‘on’ and ‘off’ states of Wnt/β-catenin signalling. Furthermore, Axin1 function has been implicated in the regulation of other major signalling cascades, including p53- and TGFβ-dependent pathways [29,30]. Based on classical protein interaction studies, a multitude of proteins may interact with Axin1 [31]. By contrast, Axin1 is present in limiting amounts in the cytoplasm, suggesting that binding partners outnumber the levels of Axin1 and that formation of Axin1-based protein complexes is highly regulated [24]. Whether Axin1 performs its roles as a scaffold protein in multiple, distinct protein complexes or rather acts in the context of large protein assemblies with multiple functions remains unknown.

Inappropriate activation of Wnt signalling is one of the most frequent signalling abnormalities in human cancer [32]. Mutations in destruction complex components such as APC and β-catenin commonly lead to the uncontrolled activation of β-catenin/TCF-mediated growth-inducing gene programmes [33,34]. Mutations in APC are responsible for familial adenomatous polyposis and occur in 85% of sporadic colorectal cancers (CRCs) [35,36]. APC is regarded as an integral component of the β-catenin destruction complex as it carries both β-catenin and Axin1 interaction domains, but its function in β-catenin turnover remains elusive. Cancer-associated APC mutations primarily comprise premature stop-codons that lead to the expression of a truncated form of the protein that lacks Axin1 interaction motifs [37]. How mutant APC affects the composition and activity of Axin1-based multi-protein complexes is unclear. Here, we analyse how Wnt stimulation or APC mutations affect the size and composition of endogenous protein complexes formed by Axin1 and β-catenin, two central components of the Wnt/β-catenin pathway. We optimized a two-dimensional electrophoresis method for simultaneous analysis of native Axin1 and β-catenin complexes at high resolution. We show that Axin1 operates in complexes of a relatively narrow size range, irrespective of Wnt signalling or APC mutations, suggesting stable interactions of Axin1 with a limited set of partners. We demonstrate that β-catenin resides in three distinct protein assemblies: (i) a high molecular weight (MW) adherens junction complex, (ii) a medium-sized Axin1-based cytosolic complex, and (iii) a low-mass cytosolic complex. The accumulation of β-catenin in small cytoplasmic complexes follows Wnt stimulation and thus can be applied to demonstrate Wnt pathway activation in unmodulated cells and tissues. Moreover, we show that phosphorylated β-catenin is released from Axin1 complexes during active signalling and contributes to the accumulating, cytoplasmic pool of β-catenin. Our results suggest that the degradation of phosphorylated β-catenin is restrained in Wnt-stimulated cells.

## 3. Results

### 3.1. Endogenous Axin1-based multi-protein complexes accommodate a limited number of binding partners

Qualitative analysis of the interactions between Axin1 and other Wnt pathway components using traditional co-immunoprecipitation experiments is well documented [3,7,8,11,28,31,38,39]. Little is known, however, about the range of complexes in which critical Wnt pathway regulators operate, how shared subunits distribute over complexes with distinct molecular activities and how the composition of these complexes is shifted during pathway stimulation.

**Figure 1.**
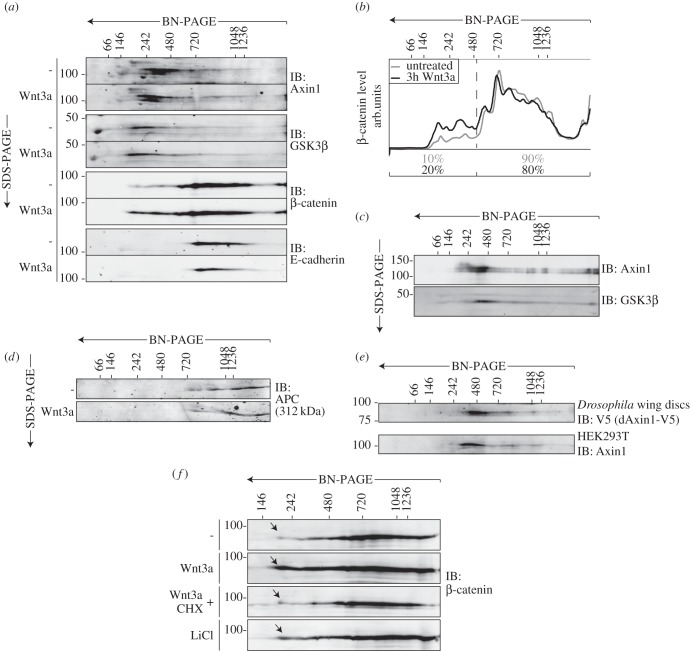
Complex formation of endogenous Wnt pathway components during signalling. (*a*) BN/SDS-PAGE analysis of Wnt pathway components in HEK293T cells that were left untreated or treated with Wnt3a-conditioned medium for 3 h. The bulk of Axin1 is present in complexes with an estimated mass of 250–750 kDa. GSK3β is present in a wide range of complexes but shows enrichment in the mass range of Axin1. β-catenin is highly enriched in complexes that co-migrate with E-cadherin complexes. Wnt stimulation induces accumulation of β-catenin in 200–500 kDa complexes, while Axin1, GSK3β and E-cadherin complexes remain unaltered. (*b*) Quantification of β-catenin levels as shown in (*a*). (*c*) BN/SDS-PAGE analysis of Axin1-Flag immunoprecipitates from HEK293T cell lysates. Axin1 and GSK3β predominantly interact in complexes of 250–500 kDa. (*d*) BN/SDS-PAGE analysis of APC complexes in HEK293T cells that were left untreated or treated with Wnt3a-conditioned medium for 3 h. The presence of APC in complexes ranging from 750 kDa to over 1.5 MDa is not altered by Wnt signalling. (*e*) BN/SDS-PAGE analysis of *Drosophila* wing discs expressing *Drosophila* Axin-V5 (DAxin-V5), driven from a transgenic BAC gene (upper panel) and of cytoplasmic Axin1 complexes of HEK293T cells (lower panel). (*f*) LiCl treatment induces formation of low mass β-catenin complexes. Induction of low mass β-catenin by Wnt3a is blocked by cycloheximide (CHX). Wnt-induced β-catenin complexes are indicated by arrows. (*a*–*f*) Protein size markers are indicated in kilodaltons.

We set out to analyse the size distribution and composition of endogenous Axin1 complexes in HEK293T cells that carry no known Wnt pathway mutations and readily induce a β-catenin-dependent transcriptional response upon stimulation with Wnt3a. To preserve native protein complexes, cells were disrupted using mild lysis buffers containing 0.1% Triton X-100, which commonly sustains the interaction of proteins during standard co-precipitation approaches. Next, protein complexes were analysed using a combination of blue native PAGE (BN-PAGE) and SDS-PAGE in a two-dimensional manner (BN/SDS-PAGE). This approach allows for the separation of multi-protein complexes ranging from 10 kDa to 2.5 MDa with higher resolution than gel filtration or sucrose density ultracentrifugation [40–42]. Intact protein complexes are first charged by Coomassie G250 and then separated according to their size (BN-PAGE). Subsequent SDS-mediated denaturation and separation (SDS-PAGE) allows for identification of individual subunits within distinct protein complexes. The results show that the majority of endogenous Axin1 forms complexes with a mass ranging from 250 to 750 kDa, while a minor fraction trails into higher MW complexes in unstimulated HEK293T cells (figure 1*a*). From these results, it follows that the vast majority of cellular Axin1 proteins, which comprise 96 kDa each, form stable interactions with only a limited number of partners at steady state.

### 3.2. Axin1, GSK3β, β-catenin and APC reside in partially overlapping complexes

Next, we analysed the size distribution of endogenous complexes formed by the Axin1-binding partners GSK3β, β-catenin and APC using BN/SDS-PAGE to estimate their level of association with Axin1. GSK3β was present in cellular complexes that range from its monomeric size of 47 kDa to complexes of over 1 MDa, and showed enriched levels in complexes between 200 and 500 kDa (figure 1*a*). The large variation in complex size is expected, as GSK3β operates in a multiplicity of complexes to regulate pathways involved in cellular differentiation, energy metabolism and cell survival [43]. We confirmed that Axin1–GSK3β complexes are formed in a mass range of 250–500 kDa using successive Axin1-Flag immunoprecipitation, elution and BN/SDS-PAGE analysis (figure 1*c*).

The vast majority (90%) of the 85.5 kDa protein β-catenin was present in complexes of 500 kDa to over 1.5 MDa (figure 1*a* and quantification in 1*b*). We postulated that this high MW β-catenin pool represents adherens junctional complexes that incorporate β-catenin via its interaction with E-cadherin [44]. Indeed, the migration pattern of E-cadherin-based protein complexes was highly similar to that of β-catenin, suggesting that E-cadherin–β-catenin interactions take place in these high MW complexes (figure 1*a*). Only a minor fraction (10%) of β-catenin migrated in complexes of lower MW (figure 1*a*,*b*).

The 312 kDa tumour suppressor APC mainly resided in high MW complexes, ranging from 750 kDa to over 1.5 MDa (figure 1*d*). Consequently, just a small fraction of APC overlaps in size with Axin1 complexes, suggesting that only a portion of APC is present in Axin1 complexes at a given time. These findings are in agreement with the multiple Wnt pathway-independent activities of APC in the control of cell division, microtubule stability and cell–cell adhesion [45–47]. Thus, as expected for the multitasking proteins GSK3β and APC, and the dual function protein β-catenin, these proteins operate in multiple protein complexes and partially overlap with each other.

### 3.3. The distribution of Axin complexes is conserved between species

We wondered whether the distribution of Axin1 complexes is conserved between species. To address this issue, we collected 150 imaginal discs from *Drosophila melanogaster* third instar larvae, in which endogenous *axin* was deleted and replaced by V5-tagged *Drosophila* Axin (DAxin). Expression of V5-DAxin was driven from a BAC transgene, allowing for endogenous expression levels. BN/SDS-PAGE analysis of wing disc lysates showed a similar distribution pattern of V5-DAxin as compared with human Axin1, with a major fraction running between 250 and 750 kDa and a smaller part trailing into higher MW regions (figure 1*e*). We conclude that the size distribution of Axin complexes is conserved between *Drosophila* and human cells.

### 3.4. Wnt induces the formation of a small-sized β-catenin complex but leaves Axin1 complexes unaffected

How does Wnt stimulation alter the formation and size distribution of Axin1-, GSK3β-, β-catenin-, E-cadherin- and APC-based multi-protein complexes? To address this issue, we stimulated HEK293T cells with Wnt3a-conditioned medium for 3 h. At this time point, Wnt signalling is acting at a full-blown level, as recognized by the induction of LRP6 phosphorylation, Axin1 dephosphorylation and β-catenin accumulation [14,20,28]. The overall size range of complexes formed by GSK3β, E-cadherin and APC remained unchanged when compared between unstimulated and Wnt-stimulated cells (figure 1*a*,*d*), suggesting that Wnt signalling does not mediate dramatic compositional rearrangements in these complexes. Notably, the size distribution of Axin1-based complexes also remained unaltered [25,26]. By contrast, Wnt stimulation induced a clear shift in the distribution of β-catenin complexes (figure 1*a*). While a large fraction of β-catenin (80%) remained present in high MW complexes that co-migrated with E-cadherin, an accumulation of β-catenin (up to 20% of total levels) was evident in a smaller sized pool of 200–500 kDa (figure 1*a* and quantification in 1*b*). Similarly, treatment of cells with LiCl, a well-known inhibitor of GSK3β and potent activator of β-catenin-mediated transcription [48], induced the formation of small-sized β-catenin complexes (figure 1*f*).

We hypothesized that the newly formed, small-sized pool of β-catenin could arise through its controlled release from existing E-cadherin-based complexes or from newly synthesized β-catenin [49]. To address this issue, we treated cells with Wnt3a and cycloheximide (CHX) to block protein synthesis. These conditions prevented Wnt-induced formation of the small-sized β-catenin complex while high MW complexes were preserved (figure 1*f*). These results strongly argue that the Wnt-induced low-MW β-catenin complexes comprised newly synthesized protein.

In conclusion, Wnt pathway activation does not induce major changes in the composition of Axin1-based complexes but induces formation of small-sized β-catenin complexes composed of newly translated protein.

### 3.5. Wnt pathway activation is marked by formation of two distinct cytosolic β-catenin complexes that reside in an Axin1-bound and Axin1-free form

**Figure 2.**
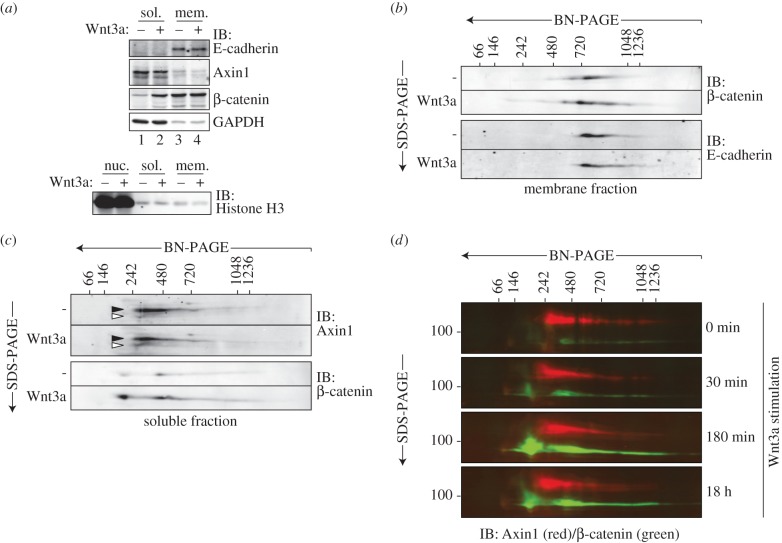
The size distribution of cytoplasmic Axin1 complexes is unaffected by Wnt signalling. (*a–c*) HEK293T cells were left untreated or treated with Wnt3a-conditioned medium for 3 h after which soluble, cytoplasmic (sol.), membrane enriched (mem.) and nuclear (nuc.) cell fractions were obtained. (*a*) Axin1 mainly resides in the cytoplasmic fraction during Wnt signalling. Cytoplasmic β-catenin accumulates in response to Wnt signalling (lanes 1 and 2), while the membrane-associated levels remain unaltered (lanes 3 and 4). Membrane and cytoplasmic protein fractions show little cross-contamination as shown by GAPDH, E-cadherin and Histone H3 immunoblots. (*b*) BN/SDS-PAGE analysis of membrane-associated β-catenin complexes during Wnt signalling. β-catenin co-migrates with E-cadherin and forms complexes of 700 kDa up to 1 MDa that remain unaffected by Wnt signalling. (*c*) BN/SDS-PAGE analysis of cytoplasmic Axin1 and β-catenin complexes during Wnt signalling. Size distribution of Axin1 complexes remains unaffected by Wnt3a stimulation (black arrowheads). Wnt3a increases the levels of a faster migrating form of Axin1 (white arrowheads). β-catenin accumulates in two distinct cytoplasmic complexes of 200 and 450 kDa in response to Wnt signalling. (*d*) BN/SDS-PAGE analysis of cytoplasmic Axin1 (red) and β-catenin (green) complexes at the indicated time points after Wnt signalling. To generate colour images, secondary antibodies with different fluorescent probes were used for immunoblotting (Alexa680 and IRDye800) and analysed with the Odyssey Infrared Imaging System. (*a*–*d*) Protein size markers are indicated in kilodaltons.

To obtain a detailed picture of the Axin1- and β-catenin-based protein complexes in untreated and Wnt-treated cells, we applied BN/SDS-PAGE analysis to cytosolic and membrane fractions of HEK293T cells. Cell fractions contained little cross-contamination as shown by immunoblotting of control proteins (figure 2*a*). As expected, Wnt stimulation induced a robust accumulation of cytosolic β-catenin on SDS-PAGE (figure 2*a*, lane 1 and 2), while the membrane pool of β-catenin remained unchanged (figure 2*a*, lane 3 and 4).

Fractionation significantly improved the resolution of the distinct protein complexes as visualized on BN/SDS-PAGE (figure 2*b*,*c*). Membrane fractions showed abundant levels of 700 kDa–1 MDa β-catenin complexes (figure 2*b*, upper panels), which co-migrated with E-cadherin (figure 2*b*, lower panels) and thus are likely to represent bona fide adherens junctional complexes. Wnt stimulation did not significantly affect the size range or intensity of the β-catenin membrane pool. These results suggest that the large pool of membrane-bound β-catenin remains in association with E-cadherin, although the Wnt-induced formation of β-catenin-containing complexes in other subcellular membrane compartments such as signalosomes or multi-vesicular bodies [14,23,28] cannot be excluded in this analysis.

In the cytosolic fraction of untreated cells, the bulk of Axin1 resided in 250–720 kDa complexes with a fraction trailing towards higher MW, matching our observations in unfractionated cell lysates (compare figure 2*c* and figure 1*a*). Wnt stimulation did not alter the overall size distribution of Axin1 complexes, but induced formation of a form that migrated faster in the second dimension (figure 2*c*, white arrowheads, 2*d* and 3*b*). This form may represent dephosphorylated Axin1, which is induced during Wnt signalling [14,50]. This hypo-modified form of Axin1 accumulated in complexes of relatively low mass compared with the total Axin1 pool, suggesting loss of one or more binding partners.

**Figure 3.**
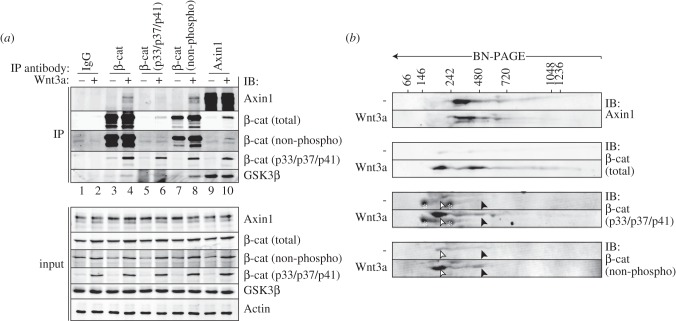
Phosphorylated and non-phosphorylated β-catenin accumulate in Axin1-bound and Axin1-free forms in response to Wnt. (*a*) Co-immunoprecipitation of Axin1 and different modified forms of β-catenin. Binding of phosphorylated (β-cat p33/p37/p41) and non-phosphorylated β-catenin (β-cat non-phospho) to Axin1 is significantly enhanced in response to Wnt signalling, while levels of associated GSK3β remain unaltered. (*b*) BN/SDS-PAGE analysis of cytoplasmic HEK293T cell fractions demonstrates that both the phosphorylated (β-cat p33/p37/p41) and non-phosphorylated (β-cat non-phospho) forms of β-catenin accumulate in small-sized complexes in the cytoplasm. Axin1-containing and Axin1-free complexes are indicated with black and white arrowheads, respectively. Protein size markers are indicated in kilodaltons. Asterisks indicate non-specific signals.

We then analysed how Wnt stimulation alters the distribution of β-catenin complexes in the cytosolic fraction. Wnt treatment induced the accumulation of β-catenin in two distinct, well-defined complexes of approximately 200 and 450 kDa, which accumulated over time during Wnt stimulation (figure 2*c*,*d*). The 200 kDa β-catenin complex runs below the lowest limit of Axin1-containing complexes. Thus, this complex represents a Wnt-induced form of β-catenin that resides separate from Axin1 in the cytosol. The 450 kDa β-catenin complex migrates within the size range of Axin1 complexes, suggesting that this pool of β-catenin resides in association with the Axin1-based destruction complex. Indeed, immunoprecipitation of endogenous Axin1 confirmed enhanced association of β-catenin in response to Wnt signalling, while levels of Axin1-associated GSK3β remained unaffected (figure 3*a*, lanes 9 and 10). In return, β-catenin precipitated higher levels of both GSK3β and Axin1 in Wnt-stimulated cells (figure 3*a*, lanes 3 and 4). Thus, our findings support the view that Axin1-based complexes accumulate β-catenin during active Wnt signalling [28]. During prolonged activation, Wnt signalling is suppressed through negative feedback mechanisms [51]. In agreement, both β-catenin accumulation and the appearance of hypo-modified Axin1 is reduced after prolonged Wnt stimulation of 18 h, when negative feedback has been established (figure 2*d*, bottom panel).

### 3.6. Phosphorylated β-catenin accumulates in both Axin1-bound and Axin1-free complexes during Wnt signalling

Capturing of β-catenin by the Axin1-based destruction complex leads to its phosphorylation and targeting for proteasomal turnover [2,8,39]. How Wnt stimulation interferes with β-catenin phosphorylation and degradation remains a subject of intense debate [14,20–28,52]. We wondered how phosphorylation alters the distribution of β-catenin complexes and how Wnt signalling interferes with these events. To address these issues, we applied antibodies that selectively recognize either the phosphorylated or unmodified GSK3β target residues on β-catenin (T41, S37 and S33). Using BN/SDS-PAGE, we observed an increase of phosphorylated and non-phosphorylated β-catenin in the 450 kDa Axin1-containing complex in cytoplasmic cell fractions (figure 3*b*, black arrowheads). These findings are in agreement with the enhanced association of β-catenin with Axin1 in Wnt-stimulated cells seen using immunoprecipitation (figure 3*a*) and by Li *et al.* [28]. In addition, we observed a striking accumulation of both non-phosphorylated and phosphorylated β-catenin in the Axin1-free 200 kDa complex (figure 3*b*, white arrowheads).

The combined results argue that phosphorylation of β-catenin continues in Wnt-stimulated cells and that phosphorylated β-catenin can be released from the Axin1 complex at this signalling stage. Consequently, kinase activity towards β-catenin must either be partially retained or restored at later time points during Wnt stimulation. Moreover, in Wnt-stimulated cells, N-terminal phosphorylation of β-catenin appears to be insufficient to cause its immediate degradation after leaving the Axin1 complex.

### 3.7. The Axin1 core complex sustains β-catenin phosphorylation in APC-mutant cells

**Figure 4.**
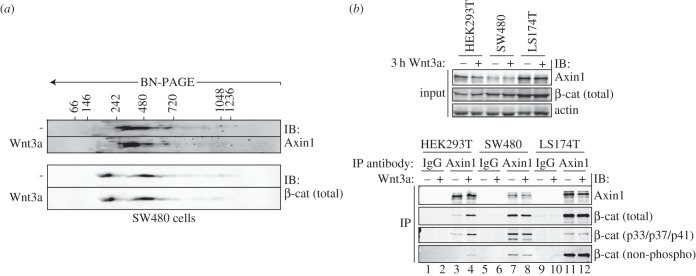
The Axin1 core complex sustains β-catenin phosphorylation in APC-mutant cells. (*a*) BN/SDS-PAGE analysis of SW480 CRC cells shows that cytoplasmic Axin1 complexes remain unaffected by APC truncation. β-catenin levels accumulate in 200 and 450 kDa complexes in these cells independent of a Wnt signal. Protein size markers are indicated in kilodaltons. (*b*) Co-immunoprecipitation of Axin1 and associated β-catenin forms in HEK293T cells (lanes 1–4) and CRC cell lines SW480 (lanes 5–8) and LS174T (lanes 9–12). Axin1 binds substantial levels of phosphorylated β-catenin in APC-truncated SW480 cells but not in β-catenin mutant LS174T cells.

The majority of colon cancer cases result from inactivating, biallelic mutations in APC, which lead to inappropriate accumulation and transcriptional activation of β-catenin [34,53]. We asked how mutant APC expression alters the distribution of Axin1 and β-catenin complexes. To address this issue, we performed BN/SDS-PAGE on cytosolic fractions of the SW480 colorectal carcinoma cell line, which expresses APC truncated at position Q1338 (figure 4*a*). The distribution of cytoplasmic Axin1 complexes in SW480 cells was very similar to that in HEK293T cells (compare figures 4*a* and 2*c*), although the trailing towards higher MW complexes appeared somewhat diminished. Strikingly, SW480 cells showed a clear accumulation of β-catenin in both 200 and 450 kDa complexes, strongly resembling the distribution pattern of β-catenin in Wnt3a-stimulated HEK293T cells (figures 4*a* and 2*c*). In further support of this observation, both non-phosphorylated and phosphorylated β-catenin accumulated within the Axin1 complex in SW480 cells as well (figure 4*b*, lanes 7 and 8). By contrast, Axin1 complexes in LS174T CRC cells, which express β-catenin carrying a mutated CK1 phosphorylation site (S45), accumulate the non-phosphorylated form (figure 4*b*, lanes 11 and 12).

In conclusion, expression of a truncated APC leaves the size range of Axin1 complexes unaffected, but leads to the accumulation of β-catenin in both Axin1-bound and Axin1-free cytosolic complexes. Moreover, the Axin1-based destruction complex still captures and phosphorylates β-catenin in APC-mutant cells. Thus, we show that the distribution of β-catenin complexes in APC-mutant cancer cells is highly similar to those of Wnt3a-stimulated cells.

### 3.8. Detection of small-sized β-catenin complexes marks Wnt pathway activation in primary human tumour cells

**Figure 5.**
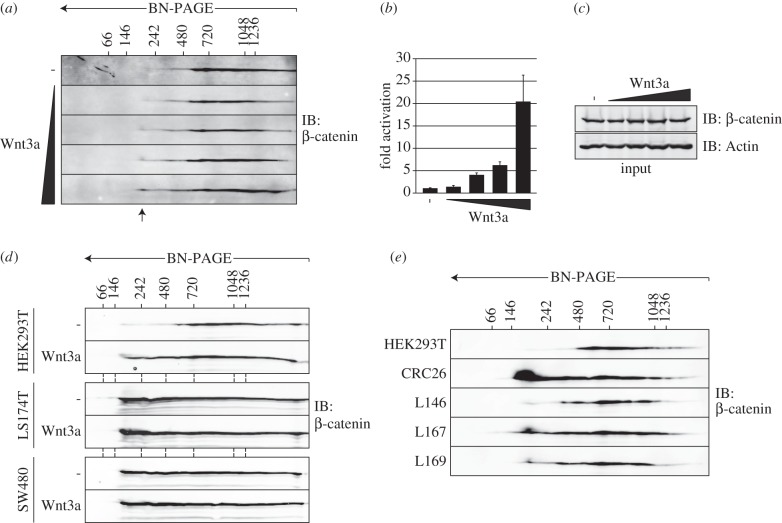
Small-sized β-catenin complexes mark Wnt pathway activation in primary human tumour cells. (*a*) Titration of Wnt3a-conditioned medium shows a dose-dependent increase in small-sized β-catenin complexes in BN/SDS-PAGE. Wnt-induced β-catenin complexes are indicated by arrows. (*b*) Wnt3a stimulation conditions shown in figure 5*a* show a dose-dependent increase of Wnt pathway activation in a TOPflash reporter assay. Relative TOPflash over FOPflash luciferase ratios (±s.d.) are shown. (*c*) Total cellular β-catenin levels remain unaffected by Wnt stimulation conditions as in (*a*,*b*). (*d*) BN/SDS-PAGE analysis of β-catenin complexes from HEK293T cells and the CRC cell lines LS174T and SW480. CRC cells accumulate high levels of low MW β-catenin complexes independent of Wnt stimulation. (*e*) BN/SDS-PAGE analysis of β-catenin complexes in untreated HEK293T cells and spheroid cultures of primary colorectal cancer (CRC26) and liver metastases (L146, L167 and L169). Low MW β-catenin complexes accumulate in the cancer-derived spheroid cultures. (*a*,*d*,*e*) Protein size markers are indicated in kilodaltons.

Our results argue that the induction of small-sized β-catenin complexes as detected by BN/SDS-PAGE could be used as a general indicator of Wnt/β-catenin pathway activation. Indeed, titration of Wnt3a-conditioned media showed a dose-dependent increase of 200–500 kDa β-catenin complexes in HEK293T cells (figure 5*a*,*b*), while the total cellular pool of β-catenin remained unaffected (figure 5*a*,*c*). In addition, the two CRC cell lines SW480 and LS174T, known to display high levels of Wnt-independent β-catenin-mediated transcription, showed accumulation of the low MW β-catenin complexes in the absence of Wnt3a (figure 5*d*).

To test the potential of BN/SDS-PAGE for assessing Wnt pathway activity in primary human tumour cells, we analysed a set of spheroid cultures of patient-derived CRC tumour cells and CRC-derived liver metastases [54], the majority of which suffer from Wnt pathway mutations [34]. In three out of four tumour samples, β-catenin was strongly enriched in 200–500 kDa complexes (figure 5*e*). These results suggest that BN/SDS-PAGE can be applied as a sensitive method to detect Wnt pathway activity in unmodulated primary tumour cells.

## 4. Discussion

The molecular steps that lead to β-catenin stabilization in Wnt-treated cells remain the subject of intense debate. One of the bottlenecks comprises the limited possibilities to study endogenous Wnt pathway proteins. Insights in the distribution of critical Wnt pathway regulators over different protein complexes is critical to understand how Wnt stimulation alters molecular interactions to drive pathway activation.

Here, we employed BN/SDS-PAGE to analyse how Wnt stimulation alters the size and composition of destruction complex components during Wnt signalling [40,42,55]. We show that protein assemblies formed by the scaffold Axin1 are of a relatively small size (250–750 kDa) in both untreated and Wnt-stimulated cells. These results suggest that the complexes in which endogenous Axin1 operates are composed of a limited number of proteins, despite the ability of Axin1 to oligomerize and associate with a broad variety of partners [31,38]. The preferred formation of multiple small complexes instead of one large assembly is partly explained by the overlap in binding sites for various partners in the Axin1 protein [31]. Importantly, the distribution pattern of Axin1 complexes is similar in *Drosophila* wing discs, indicating conservation of Axin1 complex composition. Which partners are stably incorporated in the Axin1 complex? The combined results of co-immunoprecipitation and BN/SDS-PAGE experiments confirm that the kinase GSK3β is an integral partner of the majority of Axin1 complexes, both in unstimulated and Wnt-stimulated conditions [28,56]. By contrast, APC complexes overlap only with a small fraction of Axin1 in the high MW range (over 750 kDa). The limited overlap of Axin1- and APC-based complexes is surprising, regarding the central role of APC in β-catenin destruction [7,8,33,34,57]. To our knowledge, these results provide the first quantitative information on the fraction of the total cellular Axin1 pool that engages with APC at a given time. Based on our observations, we propose that Axin1 forms stable interactions with a subset of partners, such as GSK3β, while additional factors, such as APC, may dynamically interact during certain molecular states of the Axin1 complex. Differential phosphorylation states of APC may further regulate its dynamic role in β-catenin destruction, as previously suggested [58,59].

The size distribution of the Axin1 complex was largely unaffected by Wnt signalling. These findings argue against previously proposed models in which substantial rearrangements of the Axin1-based destruction complex underlie inactivation of β-catenin destruction during Wnt stimulation [25,26]. Wnt treatment, however, did induce formation of a hypo-modified fraction of Axin1, which may correspond to a previously described Wnt-induced dephosphorylated form [14,50]. The Wnt-induced recruitment of Axin1 to the Wnt receptor complex was proposed to lead to its dephosphorylation and subsequent release in the cytosol in a conformation that is unable to bind and phosphorylate β-catenin [14]. The nature of complexes formed by dephosphorylated Axin1 in the cytoplasm, however, remains unknown. We show that hypo-modified Axin1 formed cytoplasmic complexes of a relatively small and narrow size (250–350 kDa) which, based on size-exclusion, failed to interact with β-catenin. Thus, these observations are consistent with phosphorylation-dependent regulation of the Axin1–β-catenin interaction [14]. Loss of β-catenin binding by Axin1 provides an attractive explanation for Wnt-induced inhibition of β-catenin degradation [14]. The Wnt-induced hypomodifed form of Axin1, however, represents only a small fraction of the total Axin1 pool, raising the question of how the larger part of Axin1 is affected during Wnt stimulation. Strikingly, a major fraction of Axin1 strongly accumulates β-catenin in complexes of around 450 kD, comprising both the non-phosphorylated and the phosphorylated β-catenin forms. These findings suggest that phosphorylation of β-catenin within the Axin1 complex is either partially retained or restored during Wnt signalling, as suggested previously [20,28]. Stable association of β-catenin with Axin1 in Wnt-stimulated cells was proposed to saturate and inhibit the destruction complex and prevent new cycles of β-catenin degradation [28]. Unexpectedly, we found that phosphorylated β-catenin can be released by the Axin1-GSK3β complex to accumulate in small cytosolic complexes of 200 kDa during Wnt signalling, without undergoing immediate degradation. These results are in agreement with the view that Wnt treatment inhibits the turnover of phosphorylated β-catenin, but do not support the view that β-catenin remains in tight association with the destruction complex to block entry of newly produced β-catenin [28].

What molecular steps prevent the capture and delivery of phosphorylated β-catenin to the proteasome for degradation? Some clues may come from APC-mutant cancer cells. In these cells, the size distribution of Axin1 and β-catenin complexes is highly similar to those of Wnt-stimulated cells. Moreover, phosphorylated β-catenin also accumulates in APC-mutant cells (figure 4*b*) [60]. Thus, the destruction complex does not require full-length APC *per se* to phosphorylate β-catenin. APC may rather play a role downstream of β-catenin phosphorylation in promoting the recognition and ubiquitination of phosphorylated β-catenin, as suggested previously [28,61]. Whether this molecular step is controlled by Wnt signalling remains to be determined.

Wnt stimulation leads to the accumulation of β-catenin in two cytosolic complexes of 200 and 450 kDa. Which of these complexes may feed into the nuclear, transcriptionally active β-catenin pool in Wnt-stimulated cells? As Axin1-based protein assemblies are largely retained in the cytosol [62], the 450 kDa β-catenin complex is a less likely source for transcriptionally active β-catenin. The 200 kDa β-catenin complex does not associate with Axin1 but must reside in complex with only one or two partner proteins. Furthermore, this complex is strongly accumulated in CRC cell lines and spheroid cultures in which Wnt signalling is hyperactive. Based on these findings, we consider it likely that the 200 kDa complex delivers β-catenin to the nucleus for transcriptional co-activation. The target sites for phosphorylation (Ser 33,37 and Thr 41) are located in the flexible, disordered N-terminal region of β-catenin [2,63], which is dispensable for binding to its transcriptional partners LEF1/TCF and Bcl9 [9,10,64–66]. Based on these findings, we postulate that both the phosphorylated and non-phosphorylated forms of β-catenin may contribute to target gene transcription in Wnt-stimulated cells and APC-mutant cancer cells.

In summary, we employed BN/SDS-PAGE to monitor alterations in the size distribution of critical endogenous Wnt pathway components during Wnt signalling. We show that Axin1 operates in narrowly sized complexes that do not undergo major rearrangements upon Wnt stimulation. In addition, we present supporting evidence for the Wnt-induced dephosphorylation of Axin1, which inactivates a portion of the cytoplasmic Axin1 complexes [14]. We find that Wnt-stimulated cells continue to generate phosphorylated β-catenin which accumulates in two distinct cytosolic complexes of 450 (Axin1-bound) and 200 kDa (Axin1-free). Importantly, the accumulation in Axin1-free complexes suggests that Wnt interferes with the degradation of phosphorylated β-catenin, even after it is released from the destruction complex. We thus provide evidence that both the generation and degradation of phosphorylated β-catenin are inhibited during Wnt signalling. Furthermore, our results suggest that the 200 kDa β-catenin complex provides a non-invasive and sensitive indicator of Wnt pathway activation in unmodified primary cells. Finally, we show that the distribution of β-catenin complexes in APC-mutant cancer cells is strikingly similar to that of Wnt-stimulated cells, including the accumulation of phosphorylated β-catenin. Thus, our findings support the view that APC is dispensable for β-catenin phosphorylation but rather guides later steps in β-catenin turnover. Insight in how phosphorylated β-catenin is removed from the Axin1 complex and delivered to the ubiquitin–proteasome system will be required to fully understand how cancer mutations derail Wnt signalling to drive tumour growth.

## 5. Material and methods

### 5.1. Cell culture

HEK293T and LS174T cells were cultured in RPMI medium (Life Technologies) supplemented with 10% fetal calf serum (FCS; Sigma), 100 units ml^−1^ penicillin and 100 μg ml^−1^ streptomycin (P/S; Life Technologies). Hela, MEF and SW480 cells were grown in Dulbecco's modified Eagle's medium containing 4.5 g l^−1^ glucose (Life Technologies), supplemented with 10% FCS and P/S. L-cells were grown in Dulbecco's modified Eagle's medium containing 1 g l^−1^ glucose (Life Technologies), supplemented with 10% FCS and P/S. All cells were grown at 37°C with 5% CO_2_.

### 5.2. DNA constructs and transfection

Axin1 isoform b (NM_181050) with a C-terminal Flag-tag was cloned into pcDNA4T/O (Invitrogen). HEK293T cells were transfected with FuGENE6 transfection reagent (Promega) according to the manufacturer's instructions.

### 5.3. Colorectal cancer stem cell cultures

The collection of human tumour specimens, isolation and expansion of CRC stem cell cultures were described in Emmink *et al.* [54]. Human colorectal tumour specimens were obtained from patients undergoing a liver resection for metastatic adenocarcinoma, in accordance with the medical ethical committee on human experimentation. Informed consent was obtained from all patients. All tumours were diagnosed as colorectal adenocarcinomas. Sequencing analysis of the mutational cluster region of APC showed that CRC26 cells carry bi-allelic truncations, L146 and L169 cells carry frame shifts and L167 displayed a silent polymorphism. Other genes or APC regions that might contribute to Wnt pathway deregulation were not analysed.

### 5.4. Luciferase reporter assay

HEK293T cells were seeded in 24-well plates and transfected with 5 ng TK-Renilla and 30 ng TOP-flash or FOP-flash reporter constructs. Luciferase activity was measured using the Dual Luciferase Reporter Kit (Promega), according to the manufacturer's protocol.

### 5.5. Antibodies and chemical reagents

For western blotting and immunoprecipitations, the following antibodies were used: goat anti-Axin1 (R&D Systems), rabbit anti-APC (Santa Cruz), mouse anti-β-catenin (BD Transduction Laboratories), mouse anti-GAPDH (Millipore), mouse anti-Actin (MP Biomedicals), mouse anti-β-catenin S37, T41 non-phosphorylated (Millipore), rabbit anti-phosphorylated β-catenin (pS33/pS37/pT41), rabbit anti-E-cadherin, rabbit anti-GSK3β and rabbit anti-Histone H3 (Cell Signalling Technologies). Secondary antibodies were conjugated with Alexa Fluor (Life Technologies) and IRDye (LI-COR). LiCl was used at 25 mM for 3 h. Cycloheximide was used for 4 h at 100 μM. Wnt3a-conditioned medium, derived from stably transfected L cells, was used as a supply of Wnt.

### 5.6. Immunoblotting

The Odyssey Infrared Imaging System from LI-COR was used for immunoblot analysis. β-catenin quantifications were performed with ImageJ and Odyssey v. 3.0 imaging software.

### 5.7. Blue native/SDS-PAGE

Cells were grown to 80% confluency in 10-cm dishes, scraped in PBS and pelleted. Cells were resuspended and lysed in 600 μl BN-PAGE buffer pH 7.0, containing 20 mM Bis-Tris, 20 mM NaCl, 500 mM ε-aminocaproic acid, 2 mM EDTA, 10% glycerol, 0.1% Triton X-100, 10 mM NaF, 10 mM NaVO_4_, 10 μM leupeptin, 10 μM aprotinin and 1 mM PMSF. Lysates were centrifuged at 16 000*g* for 30 min at 4°C. Supernatants were collected and subjected to buffer exchange twice using fresh BN-PAGE buffer and 30 K buffer exchange columns (Millipore). Samples were concentrated to a final volume of 60 μl and 20 μl was loaded on a 4–12% Bis-Tris nativePAGE gel (Life Technologies). NativeMark unstained protein standards (Life Technologies) were used to determine protein complex size. Gel runs were performed overnight at 22 V using NativePAGE anode and cathode buffers (Life Technologies). BN-PAGE gel slices were excised and incubated for 45 min at 70°C in two-dimensional sample buffer containing 12.5 mM Tris (pH6.8), 4% SDS, 20% glycerol, 0.02% bromophenol blue and 2.5% β-mercaptoethanol. The NativeMark gel slice was fixed and destained in 40% methanol, 10% acetic acid. Sample gel slices were placed on two-dimensional preparative SDS-PAGE gels and electrophoresis was performed at 10 mA. Western blotting was performed according to standard procedures.

### 5.8. Immunoprecipitation

Cells were grown in 10-cm dishes to 80% confluency. After washing cells with PBS, cells were scraped and lysed in lysis buffer containing 100 mM NaCl, 50 mM Tris pH 7.5, 0.25% Triton X-100, 10% Glycerol, 50 mM NaF, 10 mM Na_3_VO_4_, 10 μM leupeptin, 10 μM aprotinin and 1 mM PMSF. Lysates were cleared by centrifugation at 16 000*g* for 30 min at 4°C. For immunoprecipitation, lysates were incubated with 1 µg of antibody at 4°C for 2 h, followed by incubation with protein G beads (Millipore). For Axin1-Flag immunoprecipitations, we used 25 µl pre-coupled Flag-M2 beads (Sigma-Aldrich). Beads were washed three times with lysis buffer and twice with PBS after which samples were boiled in 1.5× SDS sample buffer. Alternatively, Flag-Axin1 immunoprecipitates were eluted in 450 µl elution buffer containing 1× TBS, 0.2% Triton X-100 and 100 µg ml^−1^ Flag Peptides (Sigma-Aldrich) at 37°C. For further analysis with BN/SDS-PAGE, the elution buffer was replaced with BN-PAGE buffer pH 7.0 using 30 K buffer exchange columns from Millipore as described above.

### 5.9. Cell fractionation

Cells were grown to 80% confluency, scraped in PBS and pelleted by centrifugation. Cell pellets were briefly washed in hypotonic lysis buffer containing 10 mM HEPES pH 7.9, 1.5 mM MgCl_2_, 10 mM KCl, 10 mM NaF, 10 mM Na_3_VO_4_, 10 μM leupeptin, 10 μM aprotinin and 1 mM PMSF. Washed cells were resuspended in fresh hypotonic lysis buffer and swollen for 30 min on ice. Cells were disrupted with a B-type Dounce. Nuclei were pelleted by centrifugation for 10 min at 500*g*. The nuclear pellet was washed in hypotonic lysis buffer once and taken up in SDS sample buffer. The supernatant was collected and centrifuged for 1 h at 100 000*g* to pellet the membrane cell fraction. Pellets were resuspended in BN-PAGE buffer and tumbled for 15 min to solubilize membrane proteins. Hereafter, insoluble cell debris was pelleted by centrifugation for 15 min at 16 000*g*. For further analysis with BN/SDS-PAGE, the buffer in the membrane and soluble protein fraction was replaced with fresh BN-PAGE buffer pH 7.0 using 30 K buffer exchange columns from Millipore as described above. The three cell fractions were taken up in a similar volume to enable quantitative comparison.

### 5.10. Generation of transgenic *Drosophila melanogaster* expressing Axin-V5

To express V5-tagged *Drosophila* Axin under the control of endogenous *axin* regulatory elements, a C-terminal V5 tag was inserted in the *axin* coding region of BAC (CH321–39B08), as described previously [67]. Insertion of the V5 tag was verified by sequencing. The modified BAC was introduced by *φ*C31-mediated integration in the VK33 (PBac y^+^-attP-3BVK00033) docking site on chromosome 3L of *D. melanogaster*. After transformants were obtained, the following genotype was recombined for this study: *BAC(AxinWT-V5) FRT82B axin^h^*/*TM6*. Exogenous Axin-V5 was confirmed to rescue deletion of endogenous *axin*.

### 5.11. Preparation of wing discs

Third instar larvae were dissected in PBS and extracted wing discs were transferred into Eppendorf tubes with ice-cold PBS. Discs were washed once with ice-cold PBS and then frozen on dry ice.
